# Current advances in regulation of bone homeostasis

**DOI:** 10.1096/fba.2020-00058

**Published:** 2020-09-19

**Authors:** Abdul Alim Al‐Bari, Abdullah Al Mamun

**Affiliations:** ^1^ Department of Pharmacy University of Rajshahi Rajshahi Bangladesh; ^2^ Department of Genetic Engineering and Biotechnology Shahjalal University of Science and Technology Sylhet Bangladesh

**Keywords:** aging, bone remodelling, cellular senescence, osteocytes, osteoporosis, RANKL‐RANK pathway

## Abstract

Bone homeostasis is securely controlled by the dynamic well‐balanced actions among osteoclasts, osteoblasts and osteocytes. Osteoclasts are large multinucleated cells that degrade bone matrix and involve in the bone remodelling in conjunction with other bone cells, osteoblasts and osteocytes, the completely matured form of osteoblasts. Disruption of this controlling balance among these cells or any disparity in bone remodelling caused by a higher rate of resorption by osteoclasts over construction of bone by osteoblasts results in a reduction of bone matrix including bone mineral density (BMD) and bone marrow cells (BMCs). The dominating effect of osteoclasts results in advanced risk of bone crack and joint destruction in several diseases including osteoporosis and rheumatoid arthritis (RA). However, the boosted osteoblastic activity produces osteosclerotic phenotype and weakened its action primes to osteomalacia or rickets. On the other hand, senescent osteocytes predominately progress the senescence associated secretory phenotype (SASP) and may contribute to age related bone loss. Here, we discuss an advanced level work on newly identified cellular mechanisms controlling the remodelling of bone and crosstalk among bone cells as these relate to the therapeutic targeting of the skeleton.

## 1 INTRODUCTION

Bone homeostasis in the adult skeleton is complex processes. Human skeletal tissue is a constant state of remodelling. The three main bone cells involve in this remodelling process‐osteoblasts, osteoclasts and osteocytes via regulation of molecular signalling pathways.^1^ In bone remodelling, the discrete zones of bone are resorbed by osteoclasts and substituted by fresh bone by osteoblasts, allowing for repair of bone micro‐injury and adapting of bone niche for control of mechanical strengths. Osteoblast cells are energetic in protein synthesis and matrix secretion to preserve and form new healthy bones. Following mineralization of bone matrix, fully differentiated and matured osteoblasts become osteocytes and are implanted in the bone matrix.^2^, ^3^ During bone remodelling process, mechanosensory cells, osteocytes act as bone orchestrators. This remodelling process is regulated by several local (e.g., growth factors, cytokines, chemokines) and systemic (e.g., oestrogens) factors that all together subscribe for bone homeostasis.^4^, ^5^ In bone modelling process, i.e., during bone development and bone resorption stages, osteocytes act autonomously to fine‐tune bone structure. Interestingly, in bone remodelling process, these cells act recycler to restore and keep skeletal health.^6^ After osteoclast‐mediated bone resorption sequence, the eroded surface of trabecular bone is engaged by osteoblasts that make bone matrix and then undertake mineralization.^7^, ^8^ Under normal physiological conditions of bone homeostasis, osteoclastic action is closely associated with osteoblastic action in such a way that the eroded bone is completely exchanged by fresh bone. Definitively, fluctuating this homeostatic equilibrium in favour of excessive osteoclast activity turns to bone pathological conditions such as osteoporosis, Paget's disease, rheumatoid arthritis (RA), osteoarthritis and autoimmune arthritis.^7^ However, imperfect osteoclast differentiation and/or function may cause osteopetrotic phenomena. On the other hand, the enhanced osteoblastic activity occurs osteosclerotic phenotype and diminished its action leads to osteomalacia or rickets. Thus, understanding the mechanism of bone homeostasis is important for the understanding of disease mechanisms and development of new therapeutics against bone diseases.

## 2 REGULATION OF BONE HOMEOSTASIS

### 2.1 Regulation of bone homeostasis by osteoclasts

**Figure 1:**
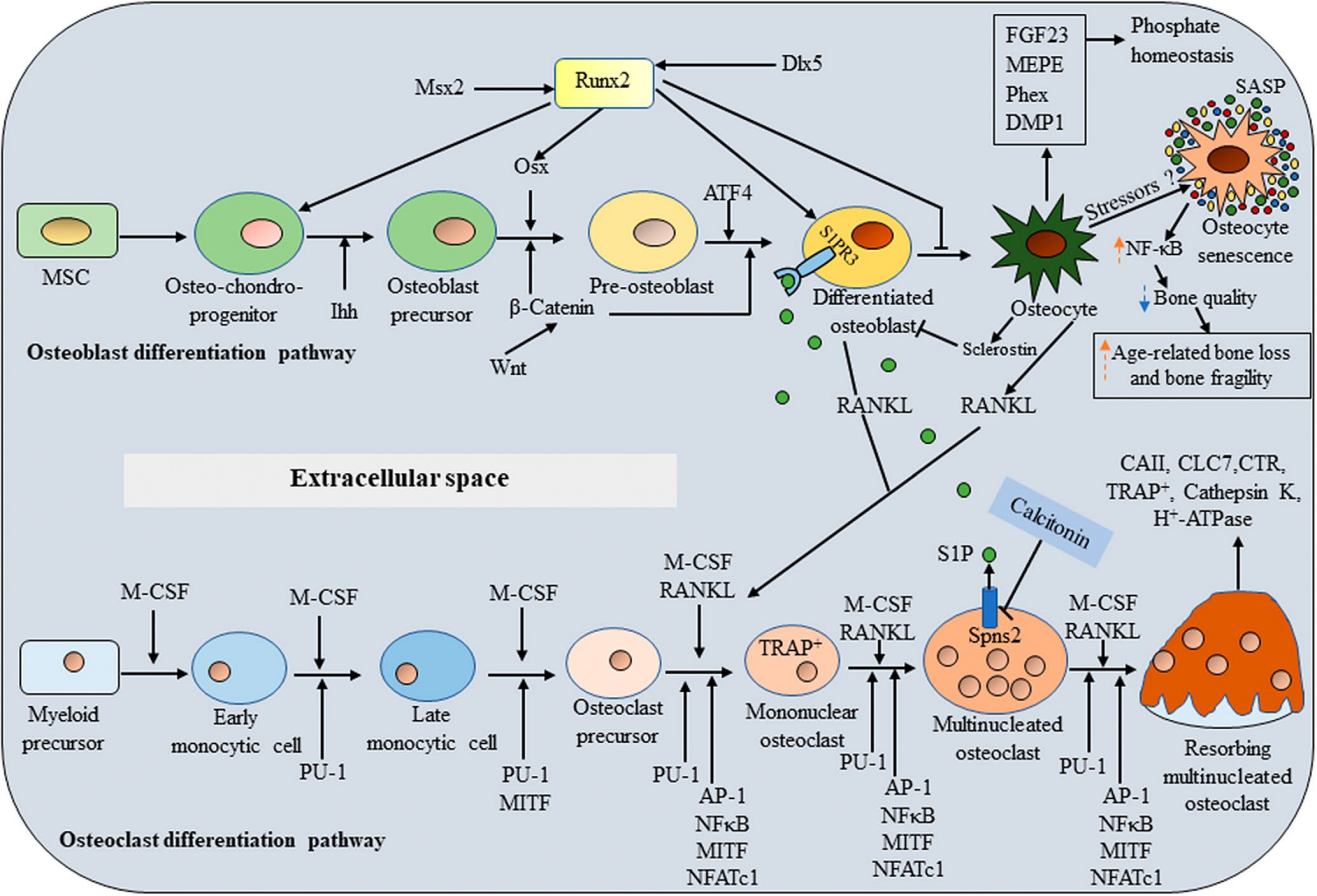
The regulation of bone homeostasis by cellular signalling molecules. Osteoblasts and chondrocytes originate from common MSC lineage precursors. Runx2, Osx, ATF4 are considered master genes for osteoblast differentiation. Ihh and Wnt/β‐catenin are also supporting signalling molecules in osteoblastogenesis. In response to various stressors such as oxidative stress, genomic instability and telomere shortening, osteocytes can be converted into osteocyte senescent cells which have SASP factor. The SASP upregulates NF‐κB and increases pro‐inflammatory factors such as IL‐1α. As a result, senescent cells and the SASP contribute to age‐related frailty and a number of age‐associated chronic diseases including osteoporosis. During osteoclast differentiation, osteoclasts derive from by the fusion of mononuclear progenitors of the monocyte/macrophage family, and osteocytes are non‐proliferative differentiated cells of the osteoblast lineage. M‐CSF and RANKL are essential external stimuli for osteoclastogenesis. PU.1, MITF, NF‐κB, AP‐1 and NFATc1 are essential for differentiation of functional osteoclasts. CT negatively regulates the osteoclast expression of spinster 2 (Spns2), which encodes a transporter for the signalling lipid sphingosine 1‐phosphate (S1P). CT suppresses Spns2 expression and reduces S1P release from osteoclasts. Sphingosine, derived from the membrane lipid sphingomyelin through intermediate ceramide, is phosphorylated to S1P by sphingosine kinases (Sphk1 and 2). S1P is either degraded by sphingosine lyase or secreted through obligatory interaction with Spns2 which is reduced by calcitonin. S1P acts through receptor S1PR3 in the osteoblast to increase osteoblast differentiation and bone formation. The osteocyte (stellate shaped blue cells on the right) expresses several membrane receptors including receptors including PTH and others and controls both osteoblast and osteoclast functions though sclerostin and RANKL. Osteocytes also secrete factors involved in phosphate homeostasis. 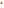 indicates upregulation and 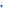 indicates down‐regulation.

**Figure 2:**
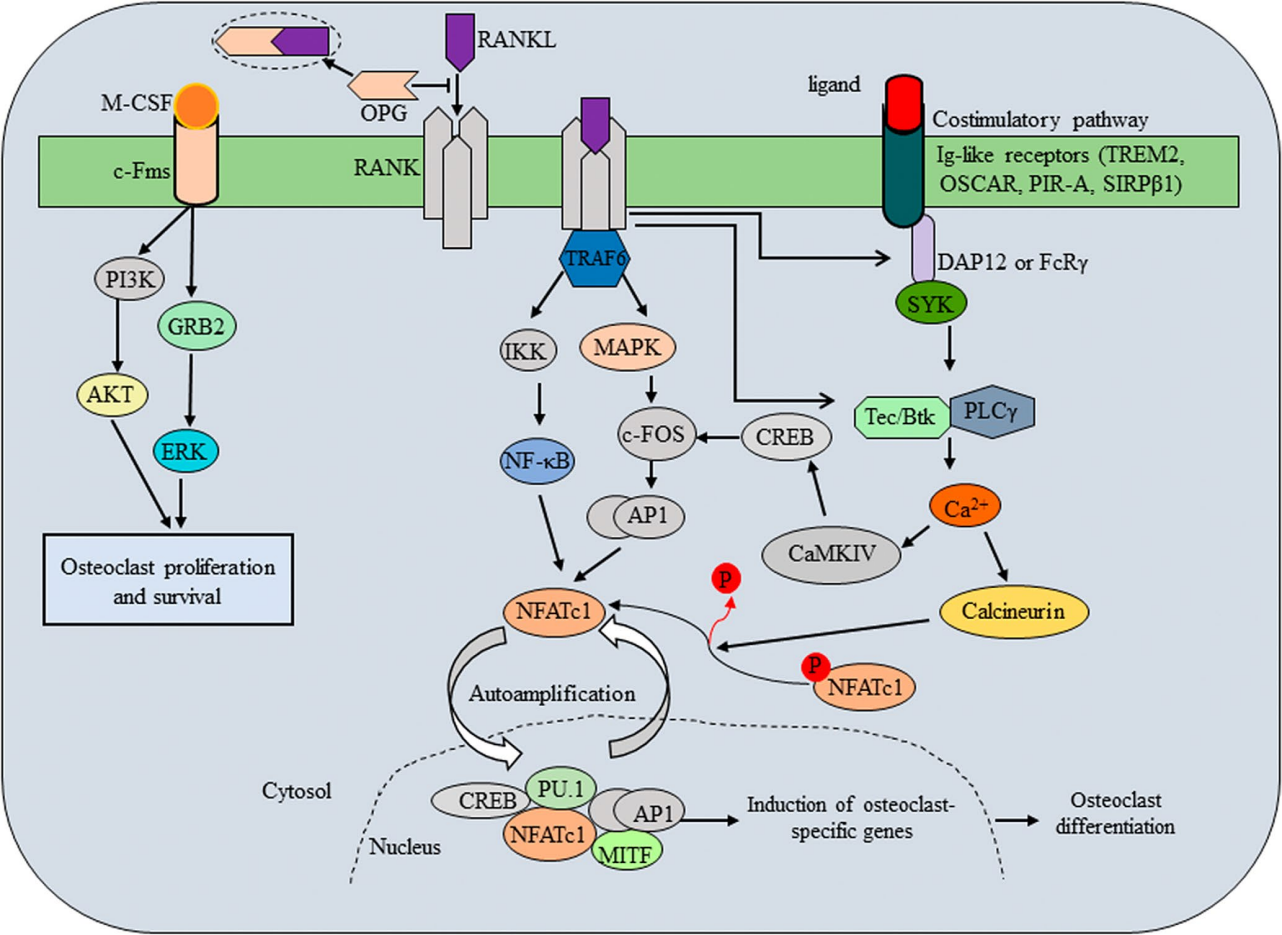
Major signaling pathways during the process of osteoclast differentiation. RANK and OPG are TNFR receptor‐related proteins, and RANKL is a TNF‐related cytokine that interacts specifically with either RANK or OPG. RANKL and M‐CSF regulate osteoclastogenesis through M‐CSF‐cFms, RANKL‐RANK as well as Ig‐like receptors associated with ITAM‐harbouring adaptor molecules such as DAP12 and Fc‐receptor common γ‐subunit (FcRγ). RANKL and its receptor RANK transduce a signal via the adaptor molecule TRAF6. TRAF6 recruits TAB2 and TAK1, which in turn activates the NF‐κB pathway and MAPK pathway. NF‐κB persuades c‐Fos expression via IKKs. Activation of MAPK pathway results in the activation of the Jun proteins. The c‐Fos and Jun proteins associate to form the complex AP‐1. The expression of the master regulator of osteoclastogenesis, NFATc1, is driven by AP‐1, NF‐κB and NFATc1 itself. The activation of NFATc1 is regulated by a co‐stimulatory signal pathway. FcRγ, DAP12 and their associating molecules activate Syk, which forms a complex with Btk/Tec and BLNK/SLP76. This complex further activates PLCγ, resulting in calcium signaling. The calcium signaling activates calcineurin, which dephosphorylates NFATc1, promoting its entry into the nucleus. Activated NFATc1 promotes its own expression making an autoamplification loop. The calcium signaling also induces c‐Fos expression via CAMKIV and CREB. NFATc1, together with other factors including PU.1 and MITF, promotes the expression of osteoclastogenic genes.

The multinucleated giant cells, osteoclasts are differentiated from osteoclast precursor cells derived from haematopoietic stem cell (HSC) niche‐monocyte/ macrophage lineage cells in presence of two essential factors: the receptor activator of nuclear factor‐κB (NF‐κB) ligand (RANKL), a tumor necrosis factor (TNF) family cytokine and the macrophage/ monocyte colony‐stimulating factor (M‐CSF) (Figure 1). M‐CSF and RANKL are predominantly expressed in osteoblasts/stromal cells and essentially regulate osteoclast differentiation and function.^9^ The M‐CSF signalling is important for osteoclast precursor cell growth and survival.^10^ M‐CSF binds to its corresponding receptor, colony stimulating factor 1 receptor (c‐Fms), present on osteoclast precursors, providing the signals for supporting the survival of these progenitors. Binding of M‐CSF to its receptor c‐Fms recruits adapter proteins and cytosolic kinases, thereby activating a variety of intracellular signals. M‐CSF also induces the receptor RANK for RANKL and other RANK/NF‐κB (nuclear factor kappa‐light‐chain‐enhancer of activated B cells) pathway components such as PI3‐kinase, the indispensable regulators for osteoclastogenesis.^11^ The RANK adopts a trimetric conformation by interacting with its extracellular signal factor RANKL. The intracellular domain of the RANK trimer is supposed to absence of signalling domains but instead, for signal transduction, recruits adaptor molecules such as TNF receptor‐associated factors (TRAFs) and Grb‐2‐associated binder (Gab) 2.^12^, ^13^, ^14^ Of the several TRAF proteins that have been associated with RANKL, including TRAF1/2/3/5/6, TRAF6 is indeed an essential adaptor required for RANK‐associated signalling for osteoclastogenesis (Figure 2). Thus, intracellular RANK signalling by its interaction with RANKL induces recruitment and activation of its adaptor TRAF6, leading to the activation of multiple downstream signalling cascades such as mitogen activated protein kinases (MAPKs) including ERK, p38, and JNK (c‐Jun N‐terminal kinases) as well as TAK1 activate inhibitory κB (IκB) kinases (IKKs) NF‐κB, Rous sarcoma oncogene (Src) and AKT (also known as protein kinase B) [11]. In addition to these two positive regulators of osteoclast differentiation, osteoblasts also express a negative regulator of osteoclast differentiation, osteoprotegerin (OPG) a secreted member of the TNF receptor superfamily.^15^ OPG inhibits osteoclastogenesis, osteoclast maturation and bone resorption by acting as a decoy receptor for both the RANK ligand (RANKL) in the RANK/RANKL/OPG axis^15^, ^16^ and tumor necrosis factor‐related apoptosis‐inducing ligand (TRAIL).^17^, ^18^ Overexpression of OPG causes osteoclast‐deficient osteopetrosis, while deletion of OPG leads to osteoporosis due to increased OC number and activity.^18^, ^19^, ^20^ TRAIL stimulates osteoclastogenesis by interacting with specific TRAIL receptors on osteoclast precursor cell surfaces, activating TRAF6 signalling, inducing receptor activator of nuclear factor‐κB (NF‐κB) signalling and upregulating nuclear factor of activated T cells cytoplasmic 1 (NFATc1) expression.^16^

Based on osteoclast gene transcription, NF‐κB and NFATc1 are the key transcription factors. NF‐κB is inactive in the cytosol in the steady state and translocates to the nucleus upon activation, which is dependent on the classical or the alternative pathways.^21^ In the classical pathway, the Iκ kinase (IKK) complex phosphorylates IκBs, the inactivators of NF‐κB. The phosphorylation of the IκB molecules leads to their ubiquitination and subsequent degradation, freeing NF‐κB. Activated NF‐κB dimers induce c‐Fos and NFATc1 expressions.^22^ Since NF‐κB and MAPK signals are not RANKL‐specific pathways and RANKL almost exclusively induce osteoclastogenesis, there are some osteoclastogenesis specific transcription factors indicated to exist. In search for such factors, it has been reported that NFATc1 plays an essential and sufficient role in osteoclastogenesis.^23^ In these cases, RANKL induces and activates NFATc1 through calcium signalling. Calcium oscillation activates calcineurin, which dephosphorylates NFATc1, enabling it to enter the nucleus, so as its expression becomes amplified. Calcium signalling is also reported inducing the c‐Fos via Ca^2+^/calmodulin‐dependent kinase (CAMK) IV‐cAMP response element‐binding protein (CREB) pathway.^24^ Activation of downstream of NF‐κB and MAPK signalling, c‐Fos and Jun proteins are dimerized to form activator protein‐1 (AP‐1). NF‐κB, AP‐1 and other transcription factors are recruited to the promoter region of the *NFATc1* after RANKL stimulation, together inducing NFATc1.^25^, ^26^, ^27^ The expression of this transcription factor is greatly upregulated after RANKL stimulation by autoamplification machinery.^21^, ^23^ At the last stage of osteoclast differentiation, NFATc1 cooperates with Fos and Jun proteins to induce osteoclast‐specific genes such as DC‐STAMP (dendritic cell‐specific transmembrane protein); OC‐STAMP (osteoclast stimulatory transmembrane protein); TRAP (tartrate‐resistant acid phosphatase), calcitonin receptor, cathepsin K and integrin β3 integrin gene^27^, ^28^, ^29^, ^30^, ^31^, ^32^, ^33^, ^34^ together with other transcription factors such as PU.1 and microphthalmia transcription factor (MITF).^26^, ^27^, ^28^, ^29^, ^30^

The RANKL signalling pathway is also coordinated co‑stimulatory signalling and calcium signalling in osteoclastogenesis. Immunoglobulin (Ig)‐like receptors are a group of proteins consisted of an extracellular region, which has an Ig‐like domain and an intracellular region, which associates with ITAM (immunoreceptor tyrosine‐based activation motif)‐ or ITIM (immunoreceptor tyrosine‐based inhibitory motif)‐harbouring adaptor proteins. (Ig)‐like receptors and their adaptors are expressed on osteoclasts as well as immune cells. FcRγ (Fc receptor common γ subunit) and DAP (DNAX‐activating protein) 12 are ITAM‐harbouring adaptors expressed in osteoclast cells that have been found to have an important role in osteoclastogenesis through NFATc1 induction.^35^ PIR‐A (paired Ig‐like receptor‐A), OSCAR (osteoclast‐associated receptor) and FcγRs (Fcγ receptors) are described to associate with FcRγ; whereas triggering receptor expressed in myeloid cells (TREM)‐2, signal‐regulatory protein (SIRP) β1, and myeloid DAP12‐associating lectin (MDL)‐1 associate with DAP12.^35^, ^36^, ^37^, ^38^ Lastly, the integrated activation of RANK‐RANKL and Ig‐like receptor signalling pathways in osteoclast differentiation are regulated by the master transcription factor, NFATc1.^21^ The activated NFATc1 expression upregulates cathepsin K, MMP9, the H^+^‐ATPase subunits and carbonic anhydrase II expressions, the vital molecules for the bone resorbing action of osteoclasts [9]. During the advanced stages of osteoclast differentiation, the several useful markers such as αvβ3 integrin and TRAP are also upregulated in associated with osteoclast maturation.^39^ These markers have been identified in osteoclasts and their precursors for facilitating the focal bone erosion in osteoporosis and rheumatoid arthritis. However, it has also been found that TRAP activity of macrophages has not substantial capability to resorb mineralized bone matrix.^40^

Calcitonin (CT), an endogenous calcium regulatory hormone, directly inhibits bone‐resorbing activity of mature osteoclasts by binding the CT receptor (CTR).^41^ It has been found that CT stops vesicular trafficking to and from the ruffled border and that it alters the cytoskeleton of the cells.^42^ CT has also been demonstrated to interfere with osteoclast differentiation from precursor cells and fusion of mononucleated precursors to form multinucleated osteoclasts in bone marrow cultures.^41^, ^43^ Indeed, the presence of the CTR is thought to be a later marker of osteoclast differentiation, and its presence is often taken as marker of a mature bone‐resorbing osteoclast.^44^, ^45^ As a pharmacologically inhibitor of bone resorption,^46^ CT is widely used therapeutic to treat metabolic bone disorders such as osteoporosis Paget's disease and malignancy‐associated hypercalcemia.^41^, ^43^, ^47^ However, it is well‐recognized that continuous CT treatment eventually causes a loss of its suppressive effects on osteoclastic bone resorption.^41^ This process is referred to as the ‘escape’ phenomenon. The mechanism of this escape phenomenon has been studied by using mouse and rat osteoclasts and the results suggest that escape or desensitization to CT is closely associated with the down‐regulation of the CT receptor (CTR)^41^ by its internalization as well as by reduced its expression of cell surface through inhibition of *de novo* CTR synthesis.^43^ The intracellular signalling responsible for CT‐induced CTR down‐regulation in mouse osteoclasts is activation of the protein kinase A pathway through cell surface CTR.^43^ However, the physiological role of CT is uncertain as mice lacking CT and CT gene‐related peptide (CGRP) have a high bone mass phenotype due to an increase in bone formation parameters.^48^ This is surprising as the CTR is expressed on osteoclasts but not on osteoblasts.^48^ To clarify the cellular and molecular mechanisms of CT action in bone remodelling, Keller and colleagues identified a novel mediator of the inhibitory effect of CT on bone formation.^49^ CT negatively regulates bone formation by inhibiting the release of the anabolic bone factor sphingosine‐1‐phosphate (S1P)^50^ a lysophospholipid from osteoclasts.^51^ CT decreases expression of *Spns2* (which encodes spinster 2, a transmembrane exporter protein for S1P) and reduce S1P levels in wild‐type but not in CTR knock‐out osteoclasts. S1P acts through its receptor S1P receptor 3 (S1PR3) in the osteoblast to increase osteoblast differentiation and bone formation.^49^, ^51^, ^52^ Thus, *Spns2* acts as an osteoclast‐secreted coupling factor that stimulates bone formation^51^ and is found to be negatively regulated by CT in wild‐type osteoclasts.

### 2.2 Regulation of bone homeostasis by osteoblasts

Adult bone marrow is known to contain mesenchymal lineage progenitors which are capable to differentiate into a range of cell lineages that form different types of tissues such as bone, cartilage and muscle.^53^, ^54^ Bone formation occurs through two developmental processes‐intramembranous and endochondral ossification. Intramembranous ossification which mainly forms craniofacial bone is mediated by the differentiation of the condensed mesenchymal stem cells (MSCs) into osteoblasts, the fundamental players for bone formation. In endochondral ossification, MSC condensation differentiates into chondrocytes, forming a cartilage template that is later replaced by bone.^55^ There are three major stages of osteoblastogenesis: proliferation, matrix maturation and mineralization. Commitment of MSCs to tissue‐specific cell types is orchestrated by several transcriptional regulators that serve as “master switches.” In skeletogenesis, the key transcription factors‐ Runx2 (Runt‐related transcription factor 2), osterix (Osx), ATF4 (activating transcription factor 4) and Dlx5 are known to play important roles in the cell‐fate decision process by which MSCs become osteoblasts through activation of cell type‐specific genes. Runt‐domain containing Runx2, a central regulator of bone formation is expressed in the condensed MSCs. Runx2 fulfils its role as a master regulatory switch through segregation of Runx2^+^cells from osteochondro‐progenitors to form precursor osteoblasts.^56^ Runx2 is also reported to have two main functions‐induction of osteogenesis and suppression of adipocyte differentiation.^2^ Although Runx2 is crucial for osteoblast differentiation, multiple genes regulate Runx2 activity and the effectiveness of Runx2 in stimulating osteoblast formation. Osx, a zinc finger‐containing transcription factor is one of the regulators of this differentiation program and acts as a down‐stream of Runx2.^2^ Osx also involves in osteogenesis, by promoting primary bone matrix formation and the commitment of MSCs to the osteogenic lineage. Thus, it is evident that Runx2 is essential to main the prechondrogenic MSCs segregate into the osteoblast precursors, whereas Osx is subsequently obligatory to complete the osteoblast differentiation pathway (Figure 1). Thus, Runx2 is called the master regulator/platform protein of osteoblast differentiation and is indispensable to bone formation.

A wide range of cytokines modulate osteoblast differentiation, including bone matrix‐derived TGF‐β (transforming growth factor beta), bone morphogenic protein 2 (BMP‐2), BMP‐4, and BMP‐7 and their inhibitors noggin, chordin and gremlin. BMP does not directly stimulate the Runx2 expression in MSCs^57^ but it helps the expression of Dlx5 (distal‐less homeobox 5) in osteoblasts^58^, ^59^ and Dlx5 then persuades expression of Runx2 in osteoprogenitor cells. Dlx5 has been found to augment Runx2 promoter activity.^58^, ^60^ Furthermore, there is cumulative evidence that Dlx5 endorses activation of osteocalcin (OCN) by establishing heterodimers with Runx2 upstream protein, Msx2 (Msh homeobox 2), a homeobox‐containing transcription factor expressed in osteoblasts during development.^61^, ^62^ ATF4 is identified by Yang et al. as a critical substrate of ribosomal protein S6 kinase polypeptide 3 (RSK2) that is obligatory for the timely commencement of osteoblast differentiation, for terminal differentiation of osteoblasts, for bone sialoprotein (BSP), and for OCN expression.^52^ Additionally, RSK2 and ATF4 post‐transcriptionally control the synthesis of type I collagen, the main constituent of the bone matrix. The study indicates that ATF4 is a critical regulator of osteoblast differentiation and function, and suggests that absence of ATF4 phosphorylation by RSK2 may part to the bone phenotype of Coffin‐Lowry syndrome.^63^

These transcription factors regulate multiple signalling pathways involved in MSC differentiation, including Ihh (Indian hedgehog), AKT, BMP, IGF and WNT/β‐catenin.^64^ Ihh and WNT/β‐catenin are key signalling molecules in osteoblastogenesis. Ihh acts in differentiation of osteoblast progenitors into Runx2^+^ osteoblast precursors. WNT/β‐catenin signalling acts later in the differentiation pathway to Osx^+^ osteoblast precursors and then to bone‐secreting osteoblasts (Figure 1).^65^ BMP2 and IGF‐1 are shown to work synergistically in the Osx upregulation.^66^ IGF‐1 is shown to promote early stages of differentiation, acting through the AKT/MAP kinase pathway, with its concentration observed to be very low in later osteogenic phenotypes.^65^ In contrast, BMP2 is observed to promote osteogenesis in a Runx2‐dependent manner, with its expression not requiring all three MAPK components (ERK, p38, and JNK), relying only on p38 and JNK. Moreover, the combined expression of BMP2 and IGF‐1 further promotes osteoblast differentiation, working through the MAPK and PKD (protein kinase D) pathways.^65^, ^66^, ^67^ In addition, alkaline phosphatase (ALP), bone sialoprotein (BSP), and collagen type 1 alpha 1 (Col1a1) are early markers of osteoblast differentiation, while parathyroid hormone (PTH)/PTH‐related peptide (PTH/PTHrP) receptor and OCN appear late, concomitantly with mineralization. Osteopontin (OPN) peaks twice, during proliferation and then again in the later stages of differentiation.^68^ In intermediate stage of osteoblast development, preosteoblasts express STRO‐1, ALP and type I collagen, and is committed to the osteoblast lineage with extensive replicative capacity, but no self‐renewal capacity.^69^ However, in the final stage of osteoblast development, the mature osteoblasts express ALP, OPN, BSP, and OCN, and lies adjacent to newly synthesized osteoid. This stage, which is responsible for the laying down of bone, has limited replicative potential.^70^ The fibroblast growth factors (FGFs) and BMP signal synergistically promote osteoblast differentiation. BMP signalling is essential for FGF‐induced osteoblast differentiation^71^ and the osteogenic effect of BMP2 is also repressed in the absence of FGF2.^72^ FGF2 is responsible for BMP‐induced nuclei translocation and accumulation of Runx2 and P‐Smad (mothers against decapentaplegic homolog)1/5/8.^73^

### 2.3 Regulation of bone homeostasis by osteocytes

Last decade has viewed a renewed curiosity in the role and biology of matrix‐embedded osteocytes and these cells have appeared as master regulators of bone homeostasis. At present, osteocytes are known to directly involve bone remodelling through the regulation of sclerostin (the product of the *SOST* gene), a WNT‐inhibitor signalling pathway that suppresses bone formation via osteoblasts^74^ and RANKL signalling pathways a cytokine required for osteoclastogenesis (Figure 1).

Canonical WNT/β‐catenin signalling is critical to regulate bone‐mass homeostasis^75^, ^76^ and occurs in an autocrine or paracrine fashion.^77^ Canonical WNT signalling is triggered by binding of extracellular WNT ligand to the low‐density lipoprotein receptor‐related protein 5/6 (LRP5/6), the transmembrane proteins, established as WNT co‐receptors and a frizzled (FZD) family cell‐surface receptor to promote formation of a ternary complex, leading to phosphorylation of the cytoplasmic domain of LRP5/6 (called activated ‘WNT on’ state).^77^ The intracellular abundance and location of β‐catenin provides a central switch in the canonical WNT signalling pathway. The LRP5/6‐WNT‐FZD ternary complex leads to translocate the destruction complex to the cell membrane, phosphorylates the intracellular region of LRP5/6, recruits AXIN protein which disrupts destruction complex activity and allows free β‐catenin to accumulate in the cytoplasm. Non‐phosphorylated β‐catenin translocates from the cytoplasm to the nucleus to regulate gene expression.^77^

Osteocytes are key players in the regulation of the canonical WNT signalling pathway as producers and targets of WNT ligands and as secretors of molecules that modulate WNT actions.^75^, ^76^ A potent antagonist of WNT signalling secreted by osteocytes is sclerostin, primarily expressed by mature osteocytes but not by early osteocytes or osteoblasts.^78^ In the WNT off (sclerostin inhibited) state, sclerostin interacts with the WNT co‐receptors LRP5/6 ^79^, ^80^ (via the first two YWTD‐EGF repeat domains) antagonizing downstream signalling.^81^, ^82^ LRP5 and LRP6 are type I transmembrane receptors (C‐terminus in cytosol) and structurally related proteins sharing around 71% homology at the nucleotide level.^83^ The structural organization of LRP4, another member of the LRP family proteins is markedly different from LRP5/6.^83^, ^84^ LRP4 is a type II transmembrane receptor (N‐terminus in cytosol) and it belongs to the LRP subfamily III along with LRP5 and LRP6.^83^, ^85^ Sclerostin also interacts with LRP4 (via the extracellular domain) which acts as a chaperone and is required for the inhibitory action of sclerostin on WNT/βcatenin signalling.^82^, ^86^, ^87^ In such ways, sclerostin prevents formation of the LRP4/5/6‐WNT‐FZD ternary complex and hence inhibits canonical WNT signalling. In the WNT off state (basal/inhibited), cytosolic β‐catenin is phosphorylated and targeted for ubiquitination by the destruction complex and degraded in the proteasome.

It has been found that calcitonin treatment significantly increases sclerostin expression in bone as well as down‐regulates two other osteocyte gene products, DMP1 (dentin matrix protein 1), MEPE (matrix extracellular phosphoglycoprotein).^88^, ^89^, ^90^ Because freshly isolated osteocytes from calvariae and long bones express *CTR* mRNA and immunohistochemistry studies reveal co‐localization of CTR and sclerostin in osteocytes in calvarial sections.^88^, ^89^, ^90^ Moreover, CTR and sclerostin expression are both lost in long‐term culture of osteocytes as well as in osteocytes declined as mice aged.^88^, ^89^ These data indicate that osteocyte‐mediated responses to CT are most likely to be of physiological relevance in young rodents. However, the mechanism by which CT might stimulate sclerostin remains undefined.

Osteocytes adjacent the endosteal surface can interconnect directly with bone marrow cells by expanding their dendrites to the marrow space and by secreting a wide variety of molecules such as sclerostin, PTHrP, prostaglandin E2 (PGE2), fibroblast growth factor‐23 (FGF23) and RANKL capable of acting locally or on distant organs. Osteocytes express also a plethora of genes required for proper matrix mineralization and phosphate homeostasis such as PHEX (phosphate‐regulating neutral endopeptidase), DMP1, MEPE as well as FGF23.^91^ Finally, these cells also synthesize a phosphoprotein, osteopontin (OPN) involved in mineralization and hematopoiesis.^92^ OPN is an HSC niche component that negatively regulates stem cell pool size. Sclerostin is highly regulated by mechanical stimuli,^93^ PTH and several other factors in humans and animals.^94^, ^95^ Osteocytes are also key regulators of bone resorption by secreting both pro‐ and anti‐osteoclastogenic factors, such as RANKL,^96^ M‐CSF and OPG.^97^, ^98^

Osteocytes also appear to be centrally involved in calcium phosphate homeostasis through vitamin D signalling pathway.^99^ Active form of vitamin D, 1α,25(OH)2D3 (also known as calcitriol) plays in bone mineralization role through binding to the vitamin D receptor (VDR) present mainly in intestine, bone, kidney, and parathyroid gland.^100^ Systemic VDR knockout mice are phenotypically very similar to those with vitamin D deficiency.^101^ In support of this notion, there is also evidence for presence of VDR transcripts in osteocytes and direct activation of 25‐hydroxyvitamin D (25(OH)D3) in osteocytes.^102^ Interestingly calcium, PTH and calcitriol increase circulating FGF23 produced primarily by osteocytes and by matured osteoblasts.^103^ FGF23 level is regulated by locally supplied bone‐derived factors including PHEX and DMP‐1 through activation of complex Klotho‐FGF‐receptor 1 and by systemic factors including calcitriol and PTH.^104^, ^105^, ^106^ Excess FGF23 in both humans and mouse models causes hypophosphatemia, suppression of calcitriol levels and rickets (in childhood) or osteomalacia (during adulthood).^103^ Elevated levels of FGF23 also cause acquired hypophosphatemic disorders, such as in tumor‐induced osteomalacia (TIO) and is an adaptive response in chronic kidney disease (CKD). In addition, increased FGF23 level affects calcitriol synthesis and degradation, thus hindering its ability to counterbalance hypophosphatemia. FGF23 impairs the production of renal calcitriol by inhibiting the expression of extra‐renal 25(OH)D3‐1α‐hydroxylase (encoded by *CYP27B1*), the enzyme that converts 25(OH)D3 to its active metabolite, calcitriol. FGF23 also upregulates the expression of cytochrome P_450_ enzyme 24‐hydroxylase (encoded by *CYP24A1*), a mitochondrial enzyme responsible for the inactivation of 25(OH)D3 and its hormonal form, calcitriol into 24‐hydroxylated products for excretion.^103^ Both of these effects of FGF23 lower circulating concentrations of calcitriol which consequently decreases the intestinal absorption of calcium phosphate. The over‐expression of *CYP24A1* leads to dysfunction of the VDR as it over metabolized the 25(OH)D3 and calcitriol. In the parathyroid, FGF23 also directly suppresses the synthesis and secretion of PTH, which indirectly contributes to suppression of calcitriol, since PTH is the primary stimulus of *CYP27B1*. Thus, CKD patients ought to experience vitamin D deficiency and subsequent osteoporosis.

### 2.4 Cellular Senescence in bone remodelling with aging

Advanced age is one of the major risk factors for osteoporosis.^107^ Cellular senescence is a common hallmark of aged tissues and is generally defined as a dynamic process whereby a cell loses its proliferative potential, undergoing an essentially irreversible growth arrest in response to various stressors such as oxidative stress, DNA damage, genomic instability and telomere shortening.^108^ Senescent cells develop a complex, altered gene expression profile that is characterized by upregulation of senescent cell antiapoptotic pathways (SCAPs).^109^ A further common feature of senescent cells is the frequent development of a distinctive pro‐inflammatory secretome, termed the senescence‐associated secretory phenotype (SASP) which comprises of cytokines, chemokines, interleukins, growth factors/regulators, and matrix‐degrading proteases.^109^ Indeed, even a relatively low abundance of senescent cells (*e*.*g*., ~10–15% in aged primates)^110^ is sufficient to cause tissue dysfunction as well as aberrant remodelling and disruption of the normal function of neighbouring tissues via secretion of their SASP.^108^, ^109^ Thus, senescent cells and the SASP are known as contributors to age‐related frailty^108^, ^109^ and a number of age‐associated chronic diseases including osteoporosis.^111^

Recently, there have been several reports describing the role of cellular senescence in the pathophysiology of osteoporosis via altered expression of SASP factors.^111^, ^112^ Senescent cells accumulate in the bone microenvironment with aging and clearance of these cells using genetic and pharmaceutical interventions improves bone quality in aged mice.^113^, ^114^ In advanced aging, multiple cell types such as containing B cells, T cells, myeloid cells, osteoprogenitors, osteoblasts and osteocytes in the bone microenvironment become senescent,^115^, ^116^ although senescent myeloid cells and senescent osteocytes predominantly develop the SASP, a pro‐inflammatory environment.^117^ The SASP has been shown to be initiated by NF‐κB^118^ and maintained in an autocrine loop, at least in part, by IL‐1α.^117^, ^119^

Given the accumulating evidence that osteocyte regulates myeloid lineage cells (eg, osteocyte RANKL production leading to osteoclast development from myeloid progenitors.^96^, ^97^ Thus, it is speculated that with aging, a subset of osteocytes become senescent and produce a SASP signal that is communicated to neighbouring myeloid lineage cells. These myeloid lineage cells may, in turn, become senescent and amplify this signal, resulting in excessive production and secretion of SASP factor, thereby creating a toxic local microenvironment that may not only lead to senescence of neighbouring cells but also contribute to age‐related bone loss. From these findings, it is clear that senescence cells act as a causal mediator of age‐related bone loss like osteoporosis.^112^, ^113^, ^117^, ^120^ Given the critical role of osteocytes in regulating bone remodelling,^117^ targeted elimination of dysfunctional senescent osteocytes can potentially reverse the SASP, enhance bone formation, and prevent age‐related bone loss. Because osteocytes are long‐lived cells that constitute more than 95% of all bone cells.^117^ For example, a Janus kinase inhibitor previously discovered to inhibit production of multiple SASP factors,^121^ ruxolitinib administration is found to induce improvements in overall bone strength in old mice.^113^ Thus, targeting senescent cells (so‐called “senotherapeutics”) may potentially aid regeneration of dysfunctional aged tissues and attractive therapeutic potential for the treatment of bone diseases like osteoporosis.

## 3 AN OVERVIEW OF EXISTING THERAPIES FOR THE TREATMENT OF BONE DISEASES SUCH AS OSTEOPOROSIS

Osteoporosis is a skeletal disease and one of the major health problems worldwide. It is characterized by degradation of bone tissue and increase in the fragility of the bones.^122^ The loss of bone mineral density (BMD) is due to the action of osteoclast cells, which are associated with modified hormone levels and factors such as ageing. Thus, rheumatic and musculoskeletal diseases like osteoporosis markedly increases the risk of skeletal fractures as well as the prevalence of comorbidities. Effective fracture prevention by reducing the loss of bone mass is the primary treatment goal for people with osteoporosis. Osteoporosis can be prevented and improved musculoskeletal health by using numerous pharmacotherapies such as biphosphonates; selective oestrogen receptor modulators (SERMs); hormone therapies; strontium ranelate; denosumab (a human monoclonal antibody with specificity for RANKL); romosozumab (a monoclonal antibody that binds to and inhibits sclerostin) or stimulating bone formation called anabolic medications e.g., PTH preparations and calcitonin therapy have been verified the effects of increased BMD and decreased risk of skeletal fractures.^123^, ^124^, ^125^ However, these treatments have some side effects, such as oily skin, fluid retention, nausea, long‐term toxicity, and even prostate cancer in males and thus natural therapies that incur better therapeutic activities and fewer side effects are hunted. Therefore, searching small molecules that precisely suppress osteoclastic action is a favourable approach of the drug discovery for the treatment and management of bone‐related diseases including osteoporosis.^126^

## 4 SUMMARY

Bone homeostasis is a dynamic equilibrium by the regulatory actions of three key bone cells, osteoclasts, osteoblasts and osteocytes. Bone homeostasis remains intact as long as the activities of these cells are well‐adjusted, and thus net bone mass is maintained. This balance implies the existence of molecular mechanisms that tightly coordinate the differentiation of osteoblasts, osteocytes and osteoclasts, as well as their migration to locations where they function. Targeting senescent cells may attractive therapeutic potential for the treatment of bone diseases like osteoporosis in the near future.

## CONFLICTS OF INTEREST

All of the authors clearly declare that they have no competing and commercial interests.

## AUTHORS’ CONTRIBUTIONS

MAAB designed the study and revised the manuscript. MAAM assisted to revise the manuscript.

## TRANSPARENCY DECLARATIONS

None to declare.
